# Clinical correlation of visuospatial evaluation with dementia diagnosis in memory clinic

**DOI:** 10.1093/geroni/igaf122.2271

**Published:** 2025-12-31

**Authors:** Rui Huang, Malini Nair, Viviana Obando, Surya Sunil, Anil Nair

**Affiliations:** ADC, Braintree, Massachusetts, United States; ADC, Braintree, Massachusetts, United States; ADC, Braintree, Massachusetts, United States; ADC, Braintree, Massachusetts, United States; ADC, Braintree, Massachusetts, United States

## Abstract

**Background:**

We attempted to find if different level of abilities to draw three-dimensional cube (stereopsis) as measured by cube-copying test can be used as an early quick screening tool for preclinical dementia diagnosis.

**Methods:**

The study was conducted at the Alzheimer Disease Center, a suburban memory clinic serving the south shore of Boston MA. We collected retrospective data by chart review on patients during the period July 2024 to March2025. A retrospective database search included subjects with memory impairment with a diagnosis code and stereopsis score. All data analyses were performed using SPSS. Pearson Correlation was performed for correlations of age and education.

**Results:**

We identified 42 subjects with stereopsis and diagnosis code. Mean Stereopsis score was 8.08 (SD 3,80) for blood Tau negative patients, 8.19 (SD 3.58) for blood Tau positive patients. Stereopsis score was statistically positively significantly related to sex (t = 2.34, p = 0.025) and education (t = 2.66, P = 0.029). Mean age was 67.5 (SD 7.32), mean education 13,64 years (SD 1.92), 45.2% Caucasians and 64.30% females. We found a insignificant association of stereopsis to the initial diagnosis code in the clinic (p = 0.896).

**Conclusion:**

Performance on the cube-copying test can be influenced by factors such as sex, education, The cube-copying test remains to potential screening tool and is not a definitive diagnostic tool for AD or other dementias and should be used in conjunction with other cognitive assessments.

